# Social Brain Perspectives on the Social and Evolutionary Neuroscience of Human Language

**DOI:** 10.3390/brainsci14020166

**Published:** 2024-02-07

**Authors:** Nathan Oesch

**Affiliations:** Department of Anthropology, University of Toronto Mississauga, Mississauga, ON L5L 1C6, Canada; nathan.oesch@stx.oxon.org

**Keywords:** social brain, language brain, social brain hypothesis, social complexity hypothesis for animal communication, social bonding, social cognitive neuroscience, language acquisition

## Abstract

Human language and social cognition are two key disciplines that have traditionally been studied as separate domains. Nonetheless, an emerging view suggests an alternative perspective. Drawing on the theoretical underpinnings of the social brain hypothesis (thesis of the evolution of brain size and intelligence), the social complexity hypothesis (thesis of the evolution of communication), and empirical research from comparative animal behavior, human social behavior, language acquisition in children, social cognitive neuroscience, and the cognitive neuroscience of language, it is argued that social cognition and language are two significantly interconnected capacities of the human species. Here, evidence in support of this view reviews (1) recent developmental studies on language learning in infants and young children, pointing to the important crucial benefits associated with social stimulation for youngsters, including the quality and quantity of incoming linguistic information, dyadic infant/child-to-parent non-verbal and verbal interactions, and other important social cues integral for facilitating language learning and social bonding; (2) studies of the adult human brain, suggesting a high degree of specialization for sociolinguistic information processing, memory retrieval, and comprehension, suggesting that the function of these neural areas may connect social cognition with language and social bonding; (3) developmental deficits in language and social cognition, including autism spectrum disorder (ASD), illustrating a unique developmental profile, further linking language, social cognition, and social bonding; and (4) neural biomarkers that may help to identify early developmental disorders of language and social cognition. In effect, the social brain and social complexity hypotheses may jointly help to describe how neurotypical children and adults acquire language, why autistic children and adults exhibit simultaneous deficits in language and social cognition, and why nonhuman primates and other organisms with significant computational capacities cannot learn language. But perhaps most critically, the following article argues that this and related research will allow scientists to generate a holistic profile and deeper understanding of the healthy adult social brain while developing more innovative and effective diagnoses, prognoses, and treatments for maladies and deficits also associated with the social brain.

## 1. Introduction

**Figure 1:**
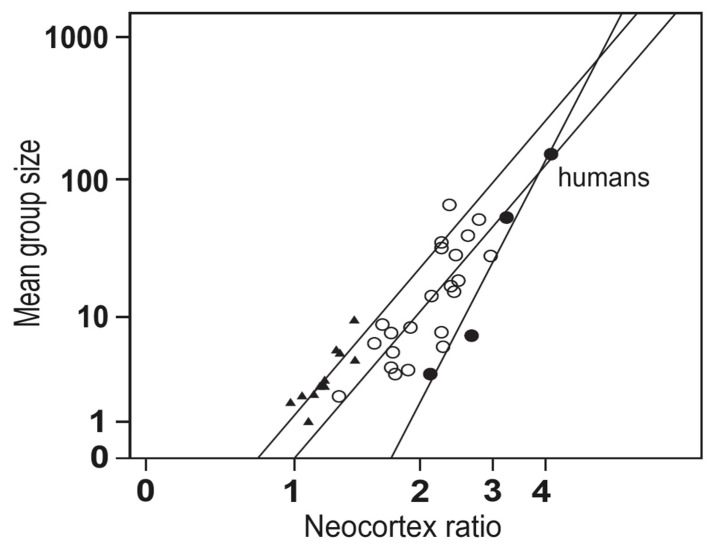
The size of the social group correlates with the encephalized size of the neocortex in anthropoid primates. Prosimians, illustrated as triangles, monkeys, illustrated as open dots, and apes, shown as solid dots, represent three distinct taxonomies in primates. Adapted from Oesch (2018) [4]. Copyright 2018 by John Wiley and Sons.

Human beings are an incredibly sophisticated species: technologically, scientifically, and cerebrally—why is this? The social brain hypothesis posits that the cognitive pressures of residing in dynamic animal societies, selected for increases in the volume of the primate brain, explain the atypically large brains of a number of anthropoid primates [1,2,3]. The initial data for this thesis came primarily from the discovery that neocortex size correlates with the size of social groupings for a variety of anthropoid primates, including humans (see Figure 1; for a review, see [4]). Since this initial finding, a substantive amount of empirical research further demonstrates that the size of the primate neocortex is associated with several distinct measures of social behavior and social cognition, including coalition frequency, deceit and deception, social learning, the prevalence of group play, female harem size, the size of grooming groups (often used to facilitate social bonding), and, of course, social group size [1]. This cognitive and behavioral complexity is partially reflected in social bonding (facilitated by grooming groups) to thwart social groupings from dissolving under these intense social dynamics.

**Figure 2:**
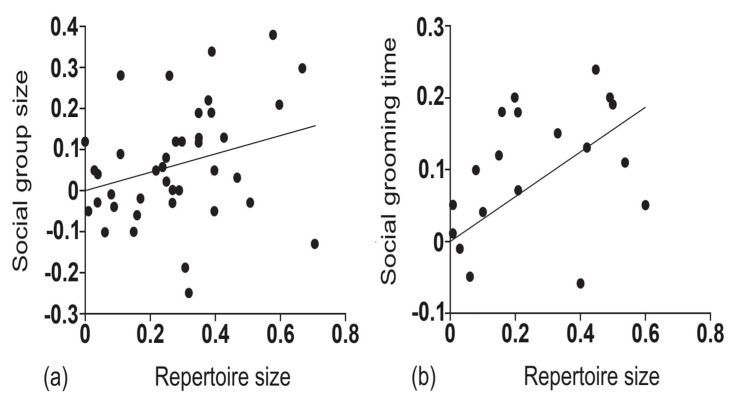
Contrasts between sister groups at every point in the phylogenetic analysis; namely, (**a**) contrasts in the size of the vocal repertoire and mean size of the social group and (**b**) contrasts in the size of the vocal repertoire and mean duration of social grooming as a metric of social bonds. Adapted from McComb and Semple (2005) [7]. Copyright 2005 by The Royal Society.

A simple extension of the social brain hypothesis, known as the social complexity hypothesis for animal communication (more informally, the social bonding hypothesis), postulates that species with complex social groupings demand complex communication systems to manage the complex social dynamics involved and promote social bonds [5,6]. The original findings in support of this thesis came from a comparative investigation of several dozen species of anthropoid primates, where both the average size of the social group and average grooming duration (as a standard metric of social bonds) were correlated with the size of the vocal repertoires of these primates (see Figure 2; [7]). Subsequent studies have further found larger vocal repertoire sizes to be correlated with long-term mating bonds among large, complex gelada baboon social groupings, compared to the much more transient matings of tinier, less complex chacma baboon groupings [8]. In humans, more recent social network analysis studies have confirmed two key predictions of the social complexity hypothesis, where group size and social density processes appear to be associated with communicative complexity and social bonding of human social networks [9,10].

Intriguingly, further support for the social bonding evolutionary function of human language, arguably the most complex of all primate communication systems, arises from sociolinguistic analyses of human conversational behavior. In particular, studies of conversational semantics reveal that, both in traditional cultures and industrial societies, gossip concerns predominate in typical dialogue, encompassing nearly 70% of daily dialogue time [11,12]. Moreover, further analyses have shown that the spread of information, relevant to personal reputation via gossip, facilitates prosocial behavior by encouraging mindful acquaintance choice when circumstances necessitate collaboration [13]. In addition, feelings of relationship closeness have also been found to be promoted via gossip, whereby the effect is often most impactful when comrades share a pessimistic perspective of an absent party [14]. Furthermore, agent-based simulation models have suggested similar conclusions, in that the evolution of communication is contingent upon an ecological requirement for large social groups, as evolutionary fitness improves as the size of the group increases, and the network attains more independent information sources [15].

In summary, the subsequent analysis examines the relationship between social cognition and language acquisition within the framework of the social brain hypothesis. This hypothesis posits that specific brain regions form a dedicated neural network for processing social information, playing a crucial role in both understanding and interacting with other individuals. Crucially, deficits within this social brain network may contribute to the language impairments observed in autism spectrum disorder (ASD). For instance, evidence will be highlighted linking mentalizing abilities—a core function of the social brain—to the development of complex syntactic structures in both typical and atypical language development. 

In particular, the following discussion reviews (1) recent developmental studies on language learning in infants and young children, pointing to the important crucial benefits associated with social stimulation for youngsters, including the quality and quantity of incoming linguistic information, dyadic infant/child-to-parent non-verbal and verbal interactions, and other important social cues integral for facilitating language learning and social bonding; (2) studies of the adult human brain, suggesting a high degree of specialization for sociolinguistic information processing, memory retrieval, and comprehension, suggesting that the function of these neural areas may connect social cognition with language and social bonding; (3) developmental deficits in language and social cognition, including autism spectrum disorder (ASD), illustrating a unique developmental profile, further linking language, social cognition, and social bonding; and (4) neural biomarkers that may help to identify early developmental disorders of language and social cognition.

## 2. The Social Brain and Social Cognitive Neuroscience

Despite the clear anthropological and evolutionary connection between social cognition, social behavior, and the social brain, described above, this framework has not yet been fully integrated into our current understanding of social cognitive neuroscience. In truth, the intricacies of the neurological computations that underpin group living in primates are substantial, including activities like coalition formation, tactical deception, organizing grooming cliques, social play, and social learning [1]. In humans, a complex network of brain regions underlies important social activities, including the recognition and cognitive processing of social signals, recognizing faces, evaluating mental states (i.e., mentalizing or theory of mind), perceiving emotions, sharing attention, determining friends from foes, evaluating others’ perceptions and beliefs, social learning, relationship formation, and social bonding [1,16,17]. 

**Figure 3:**
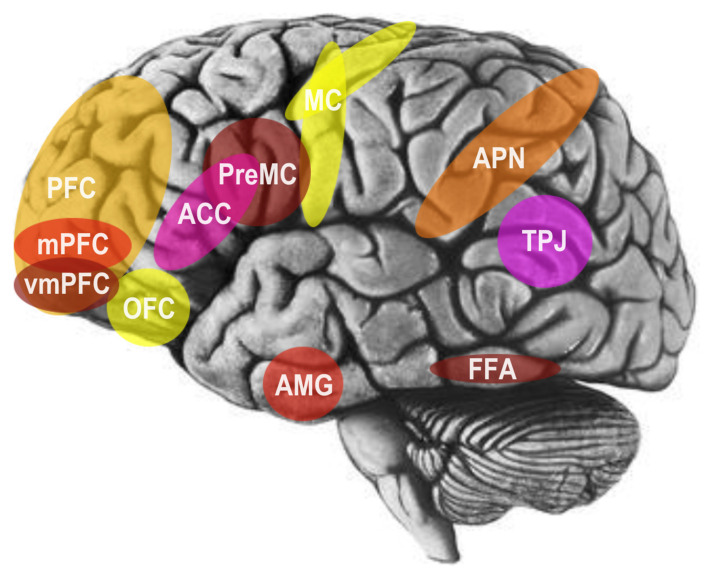
Areas of the social brain include the amygdala (AMG), anterior cingulate cortex (ACC), prefrontal cortex (PFC), fusiform face area (FFA), and temporal and parietal lobes. This includes the medial prefrontal cortex (mPFC), ventromedial prefrontal cortex (vmPFC), and orbitofrontal cortex (OFC) of the PFC, motor cortex (MC) and premotor cortex (PreMC) of the parietal lobe, fusiform face area (FFA) of the temporal lobe, and temporal-parietal junction (TPJ) and action-perception network (APN) of the temporal and parietal lobes. Adapted image from the public domain.

In a preliminary, noteworthy model of the social brain in the 1990s, neuroscientist Leslie Brothers [18] highlighted the contributions of the amygdala, orbitofrontal cortex (OFC), superior temporal sulcus (STS), and fusiform gyrus (FFG) to social information processing. More recently, functional magnetic resonance imaging (fMRI) has provided additional recognition of an interconnected network of regions joining the parietal and temporal brain lobes to the prefrontal brain lobes [4]. In particular, these include the parietal association cortex, OFC, dorsolateral prefrontal cortex, amygdala, anterior cingulate cortex (ACC), and superior temporal gyrus (STG) (see Figure 3; [16,19]).

Broadly speaking, the OFC is implicated in social reinforcement and social reward processing [16,19]. More specifically, the STS region, particularly the right-hemisphere posterior STS (pSTS) area, processes biological motion signals, like the hand, eye, and salient motions of the body, to predict and interpret the intentions and behaviors of other agents [16,19]. In addition to this area, the right inferior temporal gyrus, fusiform gyrus, right parietal lobule, and middle temporal gyrus in each hemisphere are differentially activated by processing the direction of gaze [16,19]. Several areas have been further implicated in empathy and emotional perception. For example, the amygdala has been implicated in recognizing others’ emotional states through facial expression processing and analysis, as well as in the regulation and experience of internal emotional states [16,19]. Furthermore, the FFG houses an area referred to as the fusiform face area (FFA), which is implicated specifically in the detection and recognition of faces (see Figure 3; [16,19]).

Moreover, the default mode network (DMN)—comprised of the dorsal medial prefrontal cortex (mPFC), posterior cingulate cortex, precuneus, angular gyrus, and, occasionally, right temporoparietal junction (rTPJ)—which is known for activation when an individual is unfocused on the external world and the brain is at conscious rest, further appears to be active when an individual is thinking about the self, the past and future, and most intriguingly, evaluating the mental states of other people (i.e., mentalizing or theory of mind) [20]. Further, much work now reveals that the social brain hypothesis explains not only variation in the volume of the brain between various primates, but also individual differences in the volume of the brain in humans, in regard to several different features of human social networking and social cognition. In particular, the volume of gray matter in the OFC, ACC, ventromedial prefrontal cortex (vmPFC), amygdala, and STS are associated with individual differences in higher-order intentionality capacity (i.e., advanced mentalizing or theory of mind) and social network size [21,22,23]. Lastly, recent findings from brain lesion investigations have revealed that general intelligence, emotional intelligence, and social problem-solving are underpinned by a substantively shared network of temporal, frontal, and parietal areas of the brain, including white-matter tracts connecting the areas into an organized system [24].

Lastly, recent studies of ‘mirror neurons’—neurons in the brain that activate when an organism acts, as well as when the same organism observes this same action done by another—have been postulated to be integral for mentalizing or theory of mind, language, empathy, comprehending the intentions and acts of agents, and imitative learning [25,26]. In other words, studies of the default mode neural network, especially, in studies of adult monkeys, suggest that observing an action and producing the same action oneself are neurally equivalent, and, at least in monkeys, this capacity appears to occupy a role in social comprehension and imitation [25,26]. Though mirror neurons of the brain have been observed directly in non-human primates—most notably, in macaques—in humans, brain activity merely consistent with mirror neurons has been found in the primary somatosensory cortex, inferior and superior parietal lobes, inferior frontal cortex, premotor cortex, and supplementary motor region [27]. In summary, the cognitive neuroscience of the human brain suggests a large amount of functional specialization for social perception and social information processing, including regulation from the neural network level to the neurotransmitter level, including distinctly social neurotransmitters such as oxytocin and endorphins [4,19,28,29,30,31,32].

## 3. The Social Brain and Cognitive Neuroscience of Language

In a similar fashion, despite the clear anthropological and evolutionary connection between the social brain and social communication, as described above, this framework has not yet been fully integrated into our current understanding of the cognitive neuroscience of human language [33,34]. Perhaps most critically, a complex neurological system of communication—for regulating interactions and social bonding with important members of the group—appears to be crucial for many non-human primates, including human social relationships [6]. In humans, a complex network of brain regions underlies the processing of language, including speech comprehension and production, and substantive integration with the social brain, including social-semantic working memory, and encompassing regulation from the neural network level to the neurotransmitter level, including social neurotransmitters such as oxytocin, endorphins, and dopamine [35,36,37,38,39].

**Figure 4:**
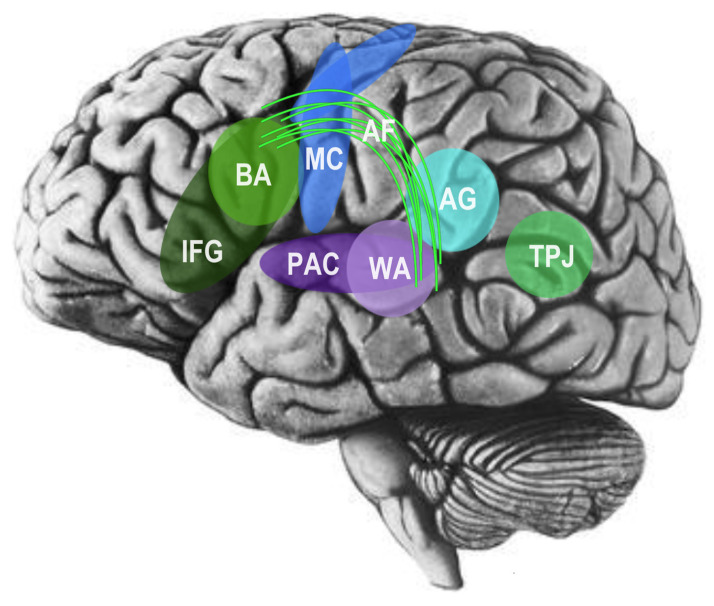
Broca’s area (BA) is related to both speech and the mirror neuron system, suggesting overlap between the networks for language, social cognition, and related social brain networks, including the inferior frontal gyrus (IFG), angular gyrus (AG), primary auditory cortex (PAC), and motor cortex (MC). The arcuate fasciculus (AF), a white-matter association tract, connects Broca’s area (BA) with Wernicke’s area (WA), a region critical for language comprehension. Canonical social brain areas, namely, the temporoparietal junction (TPJ) and left temporal lobe, appear to be associated with social-semantic working memory, further suggesting substantive overlap and integration with the language and social brain. Adapted image from the public domain.

In an influential and noteworthy model of the cognitive neuroscience of language, Pierre Paul Broca determined in 1861 that language processing areas are located primarily in the left cerebral hemisphere of the brain [40]. In later years, much research, including neuroanatomical analyses by Geschwind and Galaburda, further suggested left hemisphere dominance in brain areas dedicated to language [41,42], including myelinated axons and larger pyramidal neurons in the left hemisphere, allowing for more rapid and efficient processing of linguistic information [43,44]. Eventually, it became well-established that, at least in neurotypical adults, brain regions associated with expressive language processing, or Broca’s area, and brain regions associated with receptive language processing, also known as Wernicke’s area, are typically isolated to the left cerebral hemisphere, including white matter association tracts, such as the left arcuate fasciculus, which connect Broca’s and Wernicke’s areas into an integrated system [45,46]. But perhaps most relevantly, Broca’s area has been found to be related to both speech and the mirror neuron system, suggesting that there may in fact be substantial overlap between the neural networks for language, social cognition, and other related social brain networks (see Figure 4; [34,47,48]). 

Nonetheless, more recent work has further shown that additional areas, including the putamen, caudate nucleus, and internal capsule appear to play additional roles in language processing [49], while very young children also show significant activity in the inferior frontal and superior temporal regions of the right cerebral hemisphere—homologs of traditional left cerebral hemisphere language areas—with an activation profile in the right cerebral hemisphere that appears to diminish with age [50]. Intriguingly, homologous brain regions of Broca’s area and Wernicke’s area have also been discovered in the brains of social, group-adapted, nonhuman primates, strongly suggesting a shared evolutionary or phylogenetic history [51,52]. Though their function in nonhuman primates is poorly understood, an evolutionary perspective would suggest that they are probably central to nonhuman primate vocalization processing, in ways similar to human language processing [53,54,55,56,57,58,59].

Recent studies of the social cognitive neuroscience of language have further shown that neural activity during sentence processing in two canonical language areas, the left ventral temporoparietal junction (vTPJ) and lateral anterior temporal lobe (lATL), are associated with social-semantic working memory, in opposition to previous studies primarily implicating their role in general semantic or syntactic processing. In other words, these regions were sensitive to sentences only if the sentences conveyed social meaning. Moreover, these same regions appeared to maintain activity even after the linguistic stimuli were taken away [39]. In addition, several studies have shown that both chanting [60] and conversational speech [37] activate neural markers associated with social bonding: specifically, the temporoparietal junction (TPJ), associated with mentalizing, as well as the hypothalamus and amygdala, associated with social reward and motivation. Additional studies have revealed that self-disclosure—an important aspect of social bonding in humans—is intrinsically self-rewarding, due to activation of the mesolimbic dopamine system in the brain [38].

## 4. The Social Brain and First Language Acquisition

Historically speaking, traditional social learning theories, including those mooted by Vygotsky, Bruner, and others, have often highlighted the significance of social interaction experiences for facilitating children’s acquisition of language [61,62]. Recent empirical studies further indicate that, learning language often depends on children’s attunement to others’ responsiveness to joint visual attention, intention to communicate, and imitative impulses [61,63,64,65,66,67]. Developmental psychologists have even more recently extended these same theories and models to even earlier speech learning [68]. In particular, it has been argued that the earliest stages of language learning require social interaction; in other words, the social brain ‘gates’ the computational processes necessary for the acquisition of language [68,69,70,71]. Interestingly, randomized clinical trials appear to support this view, in finding consistent improvements in children’s expressive linguistic abilities with increases in child-to-parent interactions [72,73,74,75]. But perhaps most intriguingly, recent meta-analyses have shown that children with secure attachment to both their mother and father have greater language capabilities compared to children with one or no securely attached relationships [76].

### 4.1. Social Signals That Facilitate Early Language Acquisition

**Figure 5:**
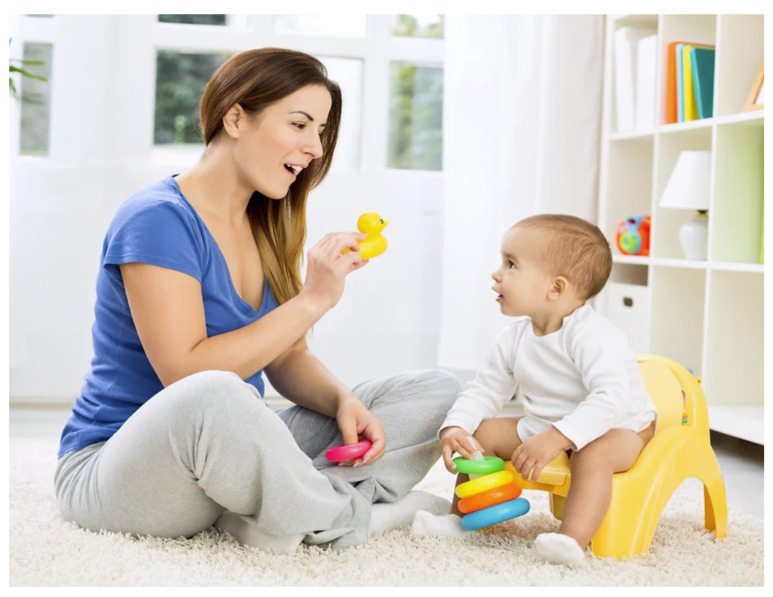
Social interaction skills, including play, reading, joint attention, and the face-to-face interactions involved in infant-directed speech (IDS) in natural language environments crucially aid the early acquisition of language. IDS aids language acquisition by providing relevant social signals (e.g., gestures, facial and emotional expressions, and directed eye gaze) that provoke infant attention and emphasize important pragmatic signals. Adapted image from the public domain.

Social interaction skills, including play, reading, reference, or joint attention between an infant or child and parent or guardian to an outward thing, and the face-to-face interactions involved in speaking in natural language environments, crucially aid the early acquisition of language (see Figure 5; [36,68,72,77,78,79,80]). In particular, infant-directed speech (IDS) and child-directed speech (CDS), or the face-to-face communication cues between an infant or child and parent or guardian, aid language acquisition by delivering relevant social signals (e.g., gestures, facial and emotional expressions, and directed eye-gaze), provoking infant attention, and emphasizing important pragmatic signals. Crucially, social interaction appears to impact the development of both speech perception and comprehension [81], as well as speech production learning [82,83,84]. For instance, in one study on speech perception, the efficacy of a live foreign-language learning social interaction was compared to the language learning efficacy of televised and audio-only presentations. Accordingly, the findings indicated a social context effect in that youngsters exposed to foreign-language learning interactions with a live human being showed robust learning effects, while linguistic stimuli delivered to infants via audio or television displayed no evidence of language acquisition [81]. In another study on speech production, infants exposed to mothers who reacted instantly to babbling by touching, smiling, and moving closer, generated more babbling than youngsters in a similarly matched control group [84].

Several important developments accompany the capacity to understand reference and joint attention of an infant and parent to an outward thing or object [68]. By 9 months of age, youngsters start to participate in individual–object–individual triadic activities in which interest is devoted to objects using gaze, which provokes attention from other individuals, also known as joint attention [65,66]. This shared perception of communicative intentions is likely to be critical for the infant’s learning of language [65,66,85], as well as understanding others as intentional agents [65,86]. Crucially, the developmental trajectory of these important social skills generally occurs simultaneously with the start of attention to linguistic units, such as phonemes, as well as later word perception, comprehension, and production (see Figure 5).

In addition, the quality and quantity of speech stimuli (e.g., vocabulary diversity, amount of word units, and mean length of utterance (MLU)) are further associated with infant vocabulary growth [87,88,89]. Unfortunately, while most language acquisition research has been conducted on families of high socioeconomic status (SES), infants raised in poorer communities with multiple challenges can affect caregiver interactions, leading to greater variability in language abilities [90,91]; although, see [92] for a recent alternative perspective.

Finally, though admittedly constituting a much smaller body of literature, at least some recent studies have noted similar effects for deaf children learning American Sign Language (ASL) [93,94,95]. For instance, at least one relevant study has noted that gaze patterns used by sign language dyads in deaf children appear to promote joint attention social behaviors, which are known from previous research to be integral for further facilitating emerging language skills [96].

### 4.2. Infant-/Child-Directed Speech and Face-to-Face Communication

Infant-directed speech (IDS) and child-directed speech (CDS) are intrinsically multimodal, and many nonverbal social signals are present during this sort of communication (e.g., gestures, facial and emotional expressions, and directed eye gaze; see Figure 5). Previous research has shown that directed eye gaze is a key form of nonverbal communication, as it facilitates language acquisition in several regards, including language processing, development of vocabulary, and perceptual mapping of form-to-object [64,97,98]. For instance, gaze following and directed eye contact provoke arousal and attention by emphasizing important social stimuli and facilitating the infant’s or child’s social engagement [99,100]. Furthermore, the capacity and willingness to engage in this sort of sustained attention predict future developments of cognition and general language skills [101,102,103]. For instance, infants who visually attend longer than infants who engage in briefer attention states demonstrate improved memory recall during object-naming tasks [104,105,106,107].

Directed gaze as a tool for language learning is typically distinguished by an early developmental trajectory where infants display a proclivity for open eyes on upright faces, involving the specialization of areas of the cortex associated with gaze processing [97,108,109,110,111]. Newborns develop the capacity for gaze following beginning from 3–4 months, becoming a consistent communication signal from 6–8 months of age [108,110]. However, it is not until 9–12 months that directed gaze begins to become an important tool used for indicating reference, facilitating language acquisition by providing directed eye gaze signaling [64,112,113,114,115]. Moreover, gaze is an important facilitator of social bonding between a mother and infant, with studies showing a positive association of maternal oxytocin to infant-to-mother gaze duration [116,117,118]. Further, directed attention toward the mouth also occupies an important part in language acquisition, delivering important cues of mouth shape and associated interpretations of speech sounds [119]. Mouth attention becomes especially pronounced in 12-month-old infants when exposed to novel words [120], non-native sounds [121], or a bilingual environment [122]. Attention to the mouth further occurs in infants from 14–18 months, coinciding with the first burst of vocabulary [123].

Quality and quantity of speech during interactions are also significant factors in language learning, especially the growth of vocabulary [124,125,126,127]. For instance, studies have shown the quantity of child-directed speech at 18 months predicts vocabulary growth at 2 years [89]. Parental engagement, namely, vocal reactions to infant vocalizations with either words or vowels, quickly influences infant vocal productions, as newborns start to assimilate phonological sound patterns spoken by the parent, facilitating the acquisition of new vocalizations [128]. Additional instances of infant-/child-directed speech and ‘parentese’ (i.e., infant-/child-directed speech with higher prosody, an enlarged vowel space, and shorter utterances [129,130,131,132]) enhance infant babbling from 6–14 months and facilitate larger vocabularies at 14 months [72]. Lastly, dyadic infant/child-to-parent verbal and non-verbal social engagement, underpinned by various social neurotransmitters, including oxytocin, serotonin, and endorphins, appears to be critical for maternal attachment and facilitating further dyadic social interactions and social bonding between a mother and an infant [28,133,134].

Child–parent social interactions are further affected by a number of environmental factors, like socioeconomic status (SES). More specifically, SES affects both the quantity and quality of parental speech stimuli [124,135]; for instance, children of low-SES families tend to display more sluggish real-time effectiveness of linguistic processing and subsequent growth of vocabulary [91]. Indeed, low SES communities and families often display significant variation in the amount of child–parent interactions, impacting the processing capability of words that are familiar, and predicting later expressive vocabulary [89]. Several factors explain the effect of low SES on social and cognitive development, including discrepancies in healthcare, sanitation, psychological and physical stress, nutrition, and environmental pollution [136]. Variability in language acquisition, as a consequence of SES, can manifest as early as 9 months of age and predict later performance in school [137,138]. 

## 5. The Social Brain, Cognitive Neuroscience of Language, and First Language Acquisition

In light of the significance of child–parent interactions for acquiring language, neuroimaging research has recently begun to investigate how just this sort of communication may impact the developing brain. As previously discussed, studies have found that, during early maturation, the *quantity* of linguistic information, as calculated by infant exposure to the number of adult words, is strongly predictive of myelin in white matter association tracts related to adult language abilities—especially the left arcuate fasciculus (AF) and superior longitudinal fasciculus (SLF) in younger children at 30 months of age—as well as youngster’s developing linguistic abilities [139]. On the other hand, the *quality* of linguistic information—word richness, dialogue experience, and mean length of utterance (MLU)—appears to be more crucial for older youth 4–6 years of age, who show greater white matter connectivity involving left AF and SLF [140,141] and greater cortical volume in the left inferior frontal gyrus (IFG) and supramarginal gyri [142], as well as older children 5–9 years of age, who show increased cortical areas in the left perisylvian areas [143]. Additional social cognitive neuroscience studies have revealed that the neural circuits underpinning the discrimination of a mother’s voice—also known as ‘motherese’, as an important component of social bonding—include voice-perception and auditory areas of the temporal lobe, reward circuit areas in the orbitofrontal cortex (OFC), nucleus accumbens (NAc), and ventromedial prefrontal cortex (vmPFC), affective processing areas, especially the amygdala, and areas related to visual face processing, especially the fusiform cortex, predict the communication and linguistic function capacities in older youth at 7–12 years of age [35]. Undoubtedly, these physiological mechanisms, also including social neurotransmitters such as oxytocin, dopamine, serotonin, and endorphins, facilitate further dyadic interactions, maternal attachment, and social bonding between a mother and infant [28,133,134].

That said, functional magnetic resonance imaging (fMRI) work on social interactions in youngsters has, until recently, primarily centered on brain activation in infants or children in reaction to a one-way social signal. However, a newly utilized technique, known as ‘hyperscanning’, allows for concurrent data collection of brain activation from multiple individuals at once, concurrently taking part in social interaction [144]. More specifically, real-time social interactions between a child and parent can be correlated with the temporal alignment of their brainwaves during such interactions. In particular, as a consequence of non-verbal and verbal signaling during social interaction, neural synchronization can occur [145,146]. Further, in at least one recent neuroimaging study, of a live two-way social interaction involving differences in speech prosody, eye gaze, and joint attention between adults and infants 9–15 months of age, distinctive paired activation occurred in infant and adult brains as a function of their social importance. For instance, activity in the prefrontal cortex in both adult and infant brains was substantively paired to the time course of two-way gaze, suggesting that agents expected joint eye contact. Greater prefrontal activation in the infant was further accompanied by variation in pitch from adult utterances, probably as a consequence of the adult’s generation of radical pitch contours in reaction to a spectrum of behaviors in youngsters, like emphasizing a particular word [147]. In short, hyperscanning imaging research presents new avenues for studying the maturation of the infant in dyadic social interactions and how a youngster’s cognitive and linguistic learning abilities may change over time.

## 6. The Social Brain and Second Language Acquisition

A relatively more recent aggregation of work has further explored the influence of bilingualism on mentalizing or perspective-taking, as well as empathy, on language processing in young children. For instance, at least one recent study found bilingual-speaking youngsters to be more accurate than monolingual speakers in a task that required analyzing an observer’s perspective from different positions [148]. Moreover, a recent meta-analysis appears to indicate these general findings are robust [149]. Though it is not fully understood how bilingualism provides this advantage, it has been suggested that bilingualism perhaps allows for additional occasions to develop executive function, metalinguistic comprehension, and improved sensitivity to the nuances of typical sociolinguistic interactions [148,149]. Further studies have suggested that empathy appears to be associated with second language learning, including pronunciation accuracy [150,151,152]. Although difficult to predict the sociological impact of this work at such an early stage in scientific development, further studies on the effects of bilingual education in young children could have important implications for academic achievement in linguistic as well as non-linguistic social cognitive areas.

## 7. The Social Brain, Developmental Dysfunctions, and Psychopathologies

Over the last few decades, increasing numbers of psychologists and neuroscientists have come to understand that many psychopathologies and developmental disorders can be largely attributed to dysfunctions of the evolved social brain [153]. In the majority of cases, such dysfunctions typically involve substantive deficiencies in social cognition, social communication, and linguistic abilities. In particular, autism spectrum disorder (ASD) is a heterogeneous disruption of social cognition, generally entailing various social deficits, such as dysfunctions in social communication (e.g., atypical facial expressions and vocal tone), social interactions (e.g., joint attention, eye gaze, and gesture), imitation and social norms, mentalizing, empathy, analogies (e.g., sarcasm and jokes), unfamiliar situations, imagination (e.g., make believe or play), and planning for or predicting future events [150]. Intriguingly, due to the profound neurogenetic and neurodevelopmental causes, as well as serious dysfunctions in social cognition that define ASD, ASD presents the occasion for neuroscientists, anthropologists, and psychologists to investigate the biological genesis of social cognition and social behaviors inherent to human nature. Additionally, numerous other psychopathologies and developmental disorders have been similarly intimated as distinct dysfunctions of social cognition and social behavior, such as borderline personality disorder, social isolation and depression, narcissistic personality disorder, bipolar disorder, schizophrenia, psychosis, and dementia. 

## 8. The Social Brain and Autism Spectrum Disorder

**Figure 6:**
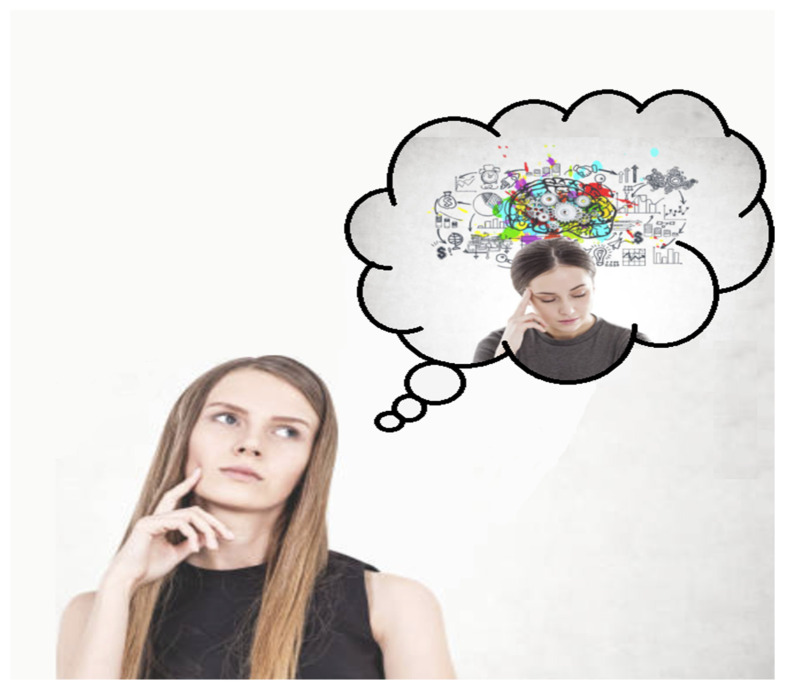
Several studies of both autistic and neurotypical adults and children appear to suggest that higher-order mentalizing (i.e., inferring the mental states of more than one individual) may be important for the syntactic ability to build subordinate and recursive embedded clauses (e.g., “Mary thinks that Sandra believes the broom is in the closet”), suggesting a direct link between social cognition and language ability. Adapted image from the public domain.

Autism spectrum disorder (ASD) is a neurodevelopmental dysfunction characterized by chronic deficits in social interaction, non-verbal and verbal communication, and social cognition, including deficits in mentalizing or the ability to understand the mental states of another individual [154,155]. Intriguingly, the complex interrelated genetic, social, and neurodevelopmental pathways and deficits found in ASD, present perhaps one of the clearest and most compelling connections between the social brain, language function, social cognition, and social bonding [19]. As the name suggests, autism is situated on a spectrum, with some individuals whose verbal capacities exist along the typical spectrum of abilities, while others never learn to speak [156]. Interestingly, in those with adequate language and cognitive capacities, such as those with Asperger’s syndrome and high-functioning autism (HFA), specifically social communicative capacities ostensibly remain impaired. In other words, communication is typically unidirectional and used instrumentally and non-socially instead of for socially related functions [157]. Neurological studies on cortical development in language-related areas of the frontal and temporal lobes of the brain have been further correlated with linguistic impairments in ASD, including asymmetrical turnaround of the frontal lobes [158,159,160], superior and anterior shifting of the left cerebral hemisphere, superior temporal sulcus, and inferior frontal sulcus [161], bilateral decreases of gray matter volume in the superior temporal sulcus [162], and apparently overall reduced left hemispheric dominance. Intriguingly, though challenging to disentwine the respective contributions of social cognition deficits in autism to linguistic deficits in autism, several recent studies in both autistic and neurotypical adults and children appear to suggest that mentalizing, which is impaired in autistic individuals, may be integral for the cognitive and linguistic ability to build subordinate and recursive embedded clauses (e.g., ‘‘Mary thinks that Sandra believes the broom is in the closet’’) (see Figure 6; [163,164,165]), suggesting another direct link between social cognition and language ability.

As previously noted, neurotypical infants and children must be attracted to and interested in infant/child-to-parent directed speech (IDS/CDS) in order to reliably acquire language. Curiously, however, youngsters with ASD generally do not prefer IDS or CDS. For instance, in one recent study, neurotypical and ASD toddlers were permitted to pick between brief ‘motherese’ spoken samples or non-spoken analogs of these same cues; however, it was solely toddlers with autism that appeared to show a preference for the nonspeech signals [166]. Moreover, the severity of ASD symptoms and the level of delay of verbal scores predicted the level of preference for nonspeech signals in children with ASD [166]. Intriguingly, this lack of interest or attention in social engagement, typical of autism, has been noted to have a profound influence on the acquisition of language, even in neurotypical cases. Among rare documented cases in which youngsters have been reared in complete isolation from social stimulation, such circumstances have had a significantly negative influence on the acquisition of language, where normal language abilities are not fully acquired [167]. Accordingly, the upshot of these studies, in neurotypical and neuroatypical adults and children, suggests that language acquisition crucially depends upon social attention to others and the social cues they generate.

## 9. Early Biomarkers of Language-Related Abilities and Relevant Clinical Applications

Describing the early development of neurotypical and neuroatypical language neurobiology is critical for the early identification and potential treatment of clinical language disorders. Crucially, delays in language and speech in infants and children can negatively affect important social and academic skills such as attention, reading, writing, social interactions, and, of course, later educational outcomes [168]. For instance, delays in language acquisition from 2–5 years are implicated in substandard reading comprehension in the classroom [169,170]. If such language delays persist after 5 years, related challenges often persist in the consequent maturation of attention, directed eye gaze, and socialization [168,171]. The majority of language delays are often noticed during parental observations or clinical check-ups when an important developmental landmark does not appear to be present, like syntactic challenges or speech onset delays. As a consequence of this rather crude ‘sit-and-wait’ approach, most youngsters are unfortunately not characterized as having had a disorder or delay of language until 2–3 years of age, which is often noted by the absence of combinatorial speech, or the capacity to formulate words into complete thoughts and sentences [168,172]. Therefore, by the time a diagnosis has been made, language delays or disorders may be magnified due to the combined effects of accumulating negative experiences, resulting in atypical development within a substandard physical and social environment. Crucially, by 3 years of age, critical neurodevelopmental milestones that support language acquisition have essentially occurred, thereby missing any opportunities for early identification and clinical intervention. Although most children eventually do come around to their age group before entering school, roughly 7–10% of children enter the classroom with chronic impairments in language development [173].

An alternative approach emphasizes the emergence of early indications, or biomarkers, of ultimate language capacities, early enough in development, to establish that any clinical interventions into speech and language delays and disorders might provide the greatest benefits. Perhaps surprisingly, there are currently no standardized or universally agreed-upon criteria in screening for language and speech deficiencies. Nonetheless, the most promising clinical interventions will most likely depend upon the very earliest identification of particular cognitive or behavioral traits, presumably underpinned by the hopeful discovery of critical neural or genetic biomarkers, which may allow for the early characterization and potential treatment of ultimate language capacities, prior to the development of language-related neurodevelopmental disorders. Biomarkers produce objective indicators of a clinical condition, evaluated reliably and accurately [174], contributing to the timely recognition of abnormal neural or behavioral patterns related to a later clinical condition. For instance, a particular pattern of brain activation at 6 months of age, could perhaps present as an informative biomarker of later pragmatic and social challenges, at a later stage of development. More broadly, biomarkers could potentially exploit the broad heterogeneity noted in ultimate language abilities, and act as reliable metrics indicative of later abnormal patterns of development. 

As might be expected, the diagnosis of language delays and disorders is usually grounded in comparable maturational landmarks observed in neurotypical language learning [175]. Children with language *delays* typically adhere to a normal maturational trajectory, albeit at more sluggish rates than would be expected [176], whereas children with language *disorders* tend to display regressions in language development (e.g., word loss from 14–21 months of age in ASD), serious and persistent delays in language learning (e.g., challenges with syntax in youngsters with specific language impairment (SLI), or impairments in at least two domains of development (e.g., such as motor function and language impairments in global developmental delay (GDD) [175,177,178]. As a general rule-of-thumb, language delays typically require clinical intervention when the development rate drops beneath 3/4 of the rate expected, for example, when a standard developmental landmark typically observed at 2 years of age fails to be met in a youngster at 30 months of age [179]. In fortunate cases, comprehensive social and language evaluations are then administered to determine whether the delayed maturational pattern(s) are associated with a primary disorder specific to language, such as SLI, or a secondary maturational disorder, like GDD or ASD. 

Nonetheless, speech and linguistic interventions should arguably begin even earlier in development. In fact, speech processing already begins in utero, in spite of the fact that the more observable first 24 months are distinguished by more obvious mappings of form-to-meaning at 5–7 months of age and proficiency at distinguishing native sounds from 6–12 months of age [180]. Unfortunately, even pediatricians and speech and language therapists with the best of intentions typically miss crucial maturational opportunities where the brain is most plastic and malleable in reaction to the environment and experience, and where the administration of targeted clinical applications could potentially deliver the most impactful therapeutic advantages.

## 10. Discussion

### 10.1. Early Biomarkers of Probable Language Outcomes and Clinical Interventions

Though biomarker approaches to language delays and disorders research remain in the earliest phases of development, recent studies in language acquisition research have pinpointed particular behavioral, cognitive, and brain metrics in first language learning utilized to evaluate ultimate language capabilities. For instance, metrics of real-time language processing abilities, especially when used in conjunction with vocabulary growth measures, appear to be a potentially promising method for recognizing ‘late talkers’ more likely to come around to normal developmental trajectories, compared to those with continual delays. For example, at least one recent study of infants 18 months of age found that both accuracy and speed metrics in a word recognition experiment predicted later variability in vocabulary learning (i.e., acceleration and rate of acquisition) in both ‘late talkers’ and typically developing children from 18–30 months of age [87]. Interestingly, this same technique further permitted the recognition of ‘late talkers’ as more probable of experiencing faster growth of vocabulary during the following 12 months. Moreover, the speed of linguistic processing efficiency, measured in 18-month-old youngsters, further appeared to predict later development in children 54 months of age on particular language measures, namely, non-verbal intelligence, receptive vocabulary, and global language abilities [181]. In a similar fashion, in high-risk SLI newborns, distinguishing particular tones at 7 months of age seems to be a significant predictor of language acquisition for language outcome measures from 1–3 years. At 3 years of age, this particular audition task, in conjunction with gender, predicts an even greater degree of performance on later language outcome metrics [182].

Additional biomarkers further demonstrate the promise of the early diagnosis, prognosis, and execution of crucial clinical mediations. In particular, neurological biomarkers of early language dysfunctions can be important for permitting clinical prognosis before relevant cognitive and behavioral symptoms emerge. For example, at least one recent study was able to use event-related potentials (ERPs: tiny voltages produced in the brain in reaction to specific events or stimuli) related to word processing in ASD children 2 years of age, to isolate early neural markers capable of indicating probable language capabilities from 4–6 years [183]. More specifically, ERP signals related to familiar words were found to be a significant indicator of adaptive behavior, cognitive ability, and receptive language measures. Perhaps surprisingly, the predictive accuracy of this metric even escalated over time, further explaining abilities at 6 years of age. Additionally, neurological biomarkers related to distinguishing native sounds are further explanatory of unique differences in later linguistic abilities; in particular, ERP signals have been found to index neural speech discrimination in infants 7 months of age, accurately predicting later language growth rates [184,185]. Moreover, these same neural patterns at 12 months further predict unique differences in spoken syntactic abilities at 6 years of age, as well as the likelihood of acquiring a more serious language or speech disorder [186]. Ultimately, the accurate recognition and isolation of early neurological biomarkers, predicting linguistic abilities, requires a deeper knowledge of the processes underpinning associations between a particular biomarker and various later language capacities. Future studies on the cognitive neuroscience of language and speech impairments should structure large sample longitudinal studies to ensure that previously identified biomarkers are consistent and reliable indicators of relevant language outcomes. In particular, studies conducted in the crucial first 24 months have the promise of isolating important neurological biomarkers and potentially predicting language disorders, delays, and abnormal neurodevelopmental trajectories. At best, this strategy can potentially promote the execution of relevant mediations at the earliest phases of development, during which language and social skills have been less impacted by experience and the brain is more moldable to crucial social and clinical mediations.

### 10.2. The Social Brain and Cognitive Neuroscience of Language

Though many behavioral studies have demonstrated the importance of synchronous speech (i.e., chanting) and other synchronous activities [187], as well as conversational dialogue for facilitating social bonding [14,188], the tools used in behavioral research are inadequate for providing a detailed understanding of the neural regions and functional brain areas underpinning synchronous speech or conversational discourse. Moreover, though chanting has been observed in every human culture, less than 5% of speech involves joint speech, and there are reasonable expectations that chanting engages additional, or even different, cortical systems than conversational speech [6,189]. Indeed, this issue is crucially important, as the mechanisms by which such vocalizations, in general, shape the developing brain are not fully understood [190]. In particular, this includes the manifestation of both pathological and healthy social development, such as autism and other disorders of social cognition, where the discernment of and social bonding to socially salient voices may be impaired. In summary, the neural mechanisms associated with social bonding during conversational speech are not well understood.

Intriguingly, Rauchbauer et al. [37] have recently shown that conversational speech activates neural markers associated with social bonding: specifically, the temporoparietal junction (TPJ), which is associated with mentalizing, as well as the hypothalamus and amygdala, which are associated with social reward and motivation. However, these results remain incomplete as they did not control for the well-documented social bonding effects of (1) conversational content, (2) eye gaze, facial expressions, and body language, (3) joint attention, and (4) voice inflection, prosody, and other emotion-related aspects of speech [6,117,191]. Although a recent study by Jasmin et al. [60] avoided many of these pitfalls, these results also remain incomplete as they primarily focused on chanting and not conversational speech. Moreover, neither Rauchbauer et al. [37] nor Jasmin et al. [60] included crucial measures of social bonding, thereby creating problems of reverse inference [192]. It thus remains unclear whether conversational speech, in general, actually facilitates social bonding or whether the social bonding effects of speech can be explained by these aforementioned potentially confounding variables. In summary, it is not well understood whether the social neuroscience of conversational discourse involves the activation of specific neural regions of interest (ROIs) related to social reward, empathy, and social bonding. Future studies on the social cognitive neuroscience of speech and social bonding should address these aforementioned concerns, as well as specifically compare neurotypical and neuroatypical participants, infants and children with adults, and male participants with female participants to determine the specific neurological profile in healthy neurotypical adults and how it varies, with specific focus on various neuroatypical social and linguistic deficits. 

### 10.3. The Social Brain and Social Complexity Hypotheses as Novel Theoretical Frameworks for Understanding the Evolution and Function of the Human Mind

In summary, important theoretical developments stemming from recent work on the social brain hypothesis (thesis of the evolution of brain size and intelligence), the social complexity hypothesis (thesis of the evolution of communication), and empirical research from comparative animal behavior, human social behavior, language acquisition in children, social cognitive neuroscience, and the cognitive neuroscience of language form a compelling framework arguing that social cognition and language are two significantly interconnected capacities of the human species, conventionally having been investigated as two separate disciplines [6]. As such, the preceding discussion has reviewed (1) recent developmental studies on language learning in infants and young children, pointing to the important crucial benefits associated with social stimulation for youngsters, including the quality and quantity of incoming linguistic information, dyadic infant/child-to-parent non-verbal and verbal interactions, and other important social cues integral for facilitating language learning and social bonding; (2) studies of the adult human brain, suggesting a high degree of specialization for sociolinguistic information processing, memory retrieval, and comprehension, suggesting the function of these neural areas may connect social cognition with language and social bonding; (3) developmental deficits in language and social cognition, including autism spectrum disorder (ASD), illustrating a unique developmental profile, further linking language, social cognition, and social bonding; and (4) neural biomarkers that may help to identify early developmental disorders of language and social cognition. Accordingly, if the current state-of-the-art in this field is any reliable indicator, additional work in this domain will likely lead to further novel, innovative, and valuable scientific developments beyond those already discussed here.

Nonetheless, despite the clear potential of this steadily emerging area of investigation, it is important to note several key limitations of the preceding review: (1) due to the relatively recent emergence of this novel, niche, and nascent discipline, the ultimate contributions this field may make in the future remain unclear; (2) due to the radically interdisciplinary nature of this area of study, consistent cross-talk and collaboration across diverse research groups may be slow in coming, and have absent focused attention and promotion, such as what the current review aims to provide; and (3) additional work relevant to these themes represents an important dearth in the current literature, especially the application of these themes related to bilingualism, sign language, and autism spectrum disorder (ASD), emphasizing the necessity of further targeted research in these areas, including work relevant to the potential interplay of bilingualism, sign language, ASD, and other neurodivergent conditions.

## 11. Conclusions

In conclusion, the human brain demonstrates a high level of specialization for social perception, social communication, language, and social information processing, including neural organization involving key brain networks and neurotransmitters, especially social neurotransmitters such as oxytocin, dopamine, serotonin, and endorphins [4,28,29,30,31,32]. Drawing on the theoretical underpinnings of the social brain hypothesis, social complexity hypothesis, and empirical research from a variety of different domains, it has been argued that social cognition and language are two significantly interconnected capacities of the human species, conventionally having been investigated as two separate disciplines [6]. In particular, studies investigating the acquisition of language suggest that infants and children benefit from many different aspects of social stimulation, depending on the specific point in the developmental timetable and during various critical periods, including the acquisition of the quality and quantity of linguistic information, dyadic infant/child-to-parent non-verbal and verbal social interactions, and other social cues for facilitating language learning and social bonding [68,80,127]. 

**Figure 7:**
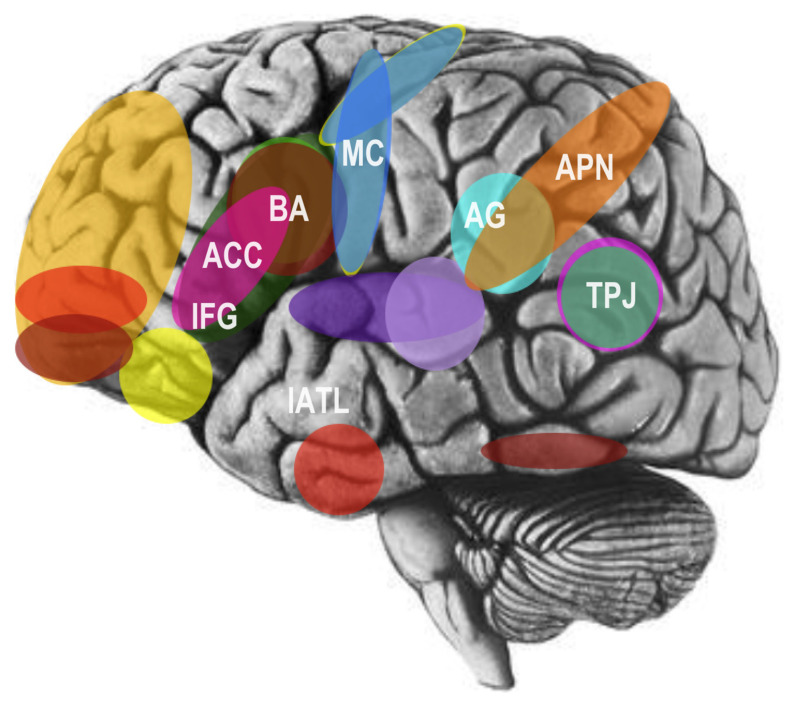
The human brain displays a high level of specialization for social and linguistic information processing. Traditional areas of the social brain are highlighted in warm colors, areas of the language brain in cool colors, pointing to substantive overlap and integration. Broca’s area (BA) is associated with both speech and mirror neurons, as the ventral temporoparietal junction (TPJ) and lateral anterior temporal lobe (lATL) are associated with social-semantic working memory, indicating the function of these areas connect social cognition with language and social bonding. Also illustrated is the inferior frontal gyrus (IFG), angular gyrus (AG), anterior cingulate cortex (ACC), action-perception network (APN) and motor cortex (MC). For simplicity, not all areas included in the networks are shown. Adapted image from the public domain.

Detailed studies of comparative animal behavior, human social behavior, profound deficits of social cognition like autism spectrum disorder (ASD), social cognitive neuroscience, and the cognitive neuroscience of language in adults suggest a similar profile. In particular, the human brain shows a high level of specialization and functional overlap of neural areas dedicated to social and linguistic memory retrieval, information processing, and comprehension, intimating the evolutionary function of these areas connects social cognition with language and social bonding (see Figure 7). In effect, the social brain and social complexity hypotheses may help to explain how neurotypical children and adults learn language, why autistic children and adults exhibit simultaneous deficiencies in language and social cognition, and why nonhuman primates and other organisms with significant computational capabilities do not develop the capacity for language [4,68]. This and related research, in conjunction with studies of early development, will allow scientists to generate a holistic profile, an understanding, and potential treatment of maladies and deficits associated with the social brain.

## Data Availability

Not applicable.
