# Correction: Oesch, N. Social Brain Perspectives on the Social and Evolutionary Neuroscience of Human Language. *Brain Sci.* 2024, *14*, 166

**DOI:** 10.3390/brainsci15030259

**Published:** 2025-02-28

**Authors:** Nathan Oesch

**Affiliations:** Department of Anthropology, University of Toronto Mississauga, Mississauga, ON L5L 1C6, Canada; nathan.oesch@stx.oxon.org

## Additional Affiliation

In the published publication [1], there was an error regarding the affiliation for Nathan Oesch. The updated affiliation should only include the following: Department of Anthropology, University of Toronto Mississauga, Mississauga, ON L5L 1C6, Canada. The authors state that the scientific conclusions are unaffected. All authors have read and agreed to the published version of the manuscript. This correction was approved by the Academic Editor. The original publication has also been updated.
